# On the p-AlGaN/n-AlGaN/p-AlGaN Current Spreading Layer for AlGaN-based Deep Ultraviolet Light-Emitting Diodes

**DOI:** 10.1186/s11671-018-2776-y

**Published:** 2018-11-08

**Authors:** Jiamang Che, Chunshuang Chu, Kangkai Tian, Jianquan Kou, Hua Shao, Yonghui Zhang, Wengang Bi, Zi-Hui Zhang

**Affiliations:** 1 Institute of Micro-Nano Photoelectron and Electromagnetic Technology Innovation, School of Electronics and Information Engineering, Hebei University of Technology, 5340 Xiping Road, Beichen District, Tianjin, 300401 People’s Republic of China; 2 Key Laboratory of Electronic Materials and Devices of Tianjin, 5340 Xiping Road, Beichen District, Tianjin, 300401 People’s Republic of China

**Keywords:** DUV LED, Current spreading, Valence band barrier height, External quantum efficiency, Wall-plug efficiency

## Abstract

In this report, AlGaN-based deep ultraviolet light-emitting diodes (DUV LEDs) with different p-AlGaN/n-AlGaN/p-AlGaN (PNP-AlGaN) structured current spreading layers have been described and investigated. According to our results, the adopted PNP-AlGaN structure can induce an energy barrier in the hole injection layer that can modulate the lateral current distribution. We also find that the current spreading effect can be strongly affected by the thickness, the doping concentration, the PNP loop, and the AlN composition for the inserted n-AlGaN layer. Therefore, if the PNP-AlGaN structure is properly designed, the forward voltage, the external quantum efficiency, the optical power, and the wall-plug efficiency for the proposed DUV LEDs can be significantly improved as compared with the conventional DUV LED without the PNP-AlGaN structure.

## Introduction

Since the first occurrence in 2003, AlGaN-based deep ultraviolet light-emitting diodes (DUV LEDs) have been attracting much interest due to their wide range of applications such as water sterilization and air purification [1–7]. However, the external quantum efficiency (EQE) for DUV LEDs is lower than 10% when the emission wavelength is shorter than 300 nm [8], which significantly limits their further application. The low EQE partially arises from the poor internal quantum efficiency (IQE). Substantial attention has been drawn that the IQE is remarkably influenced by the carrier injection and the extended dislocations [8]. AlGaN-based DUV LEDs that are grown on insulating sapphire substrates employ the flip-chip structure for the better light extraction efficiency. Nevertheless, the flip-chip DUV LED structure requires the n-electrode and the p-electrode to be on the same side. Therefore, there easily occurs the nonhomogeneous lateral current distribution, i.e., current crowding effect [9]. The current crowding effect can easily cause the local Joule heating effect and the uneven light emission [10–12]. It is worth mentioning that the local overheating seriously deteriorates the service lifetime of DUV LEDs. Moreover, the very poor Mg doping efficiency for the Al-rich p-AlGaN-based hole injection layer leads to the bad electrical conductivity [13], which further manifests the importance for improving the current spreading for DUV LEDs. Although Khan et al. have pointed out that the current crowding shall be paid attention to in their review article [14], detailed analysis regarding the current crowding and the solutions for it are less discussed for DUV LEDs until now.

Extensive techniques for promoting current spreading have been reported for GaN-based blue LEDs, and the current spreading can be improved by, e.g., selectively ion-implanting the p-GaN layer [15, 16], inserting a current blocking layer (CBL) [17–19], selectively producing nitrogen vacancies to compensate the holes in the p-GaN layer [20], optimizing the annealing process for Ohmic contact [21]. Besides using the post-fabrication approaches, the current spreading layer can also be obtained by in situ epitaxial growth in the metal-organic chemical vapor deposition (MOCVD) system. Important examples are as follows: the short-period p-GaN/i-InGaN superlattice structure between multiple quantum wells (MQWs) structure and the p-GaN layer [22, 23], the tunnel junctions [24, 25], and barrier junctions [10]. Nevertheless, reports on epi-structures to improve the current spreading for DUV LEDs can be rarely found. In this letter, we propose using p-AlGaN/n-AlGaN/p-AlGaN (PNP-AlGaN) layer to better spread the lateral current for DUV LEDs. The PNP-AlGaN structure can generate the energy barrier in the valence band of the p-type hole injection layer. The energy barrier can modulate the electrical resistivity for the p-type hole injection layer, and therefore, the current flow path can be tuned. By properly designing the PNP-AlGaN current spreading layer, the EQE, the wall-plug efficiency (WPE), and the forward voltage can be improved. Furthermore, this work also comprehensively investigates the sensitivity of the current spreading, the EQE, the WPE, and the forward voltage to the PNP-AlGaN loop, the Si doping concentration, the thickness, and the AlN composition for the inserted n-AlGaN layer of the PNP-AlGaN architecture.

## Research Methods and Physics Models

**Fig. 1:**
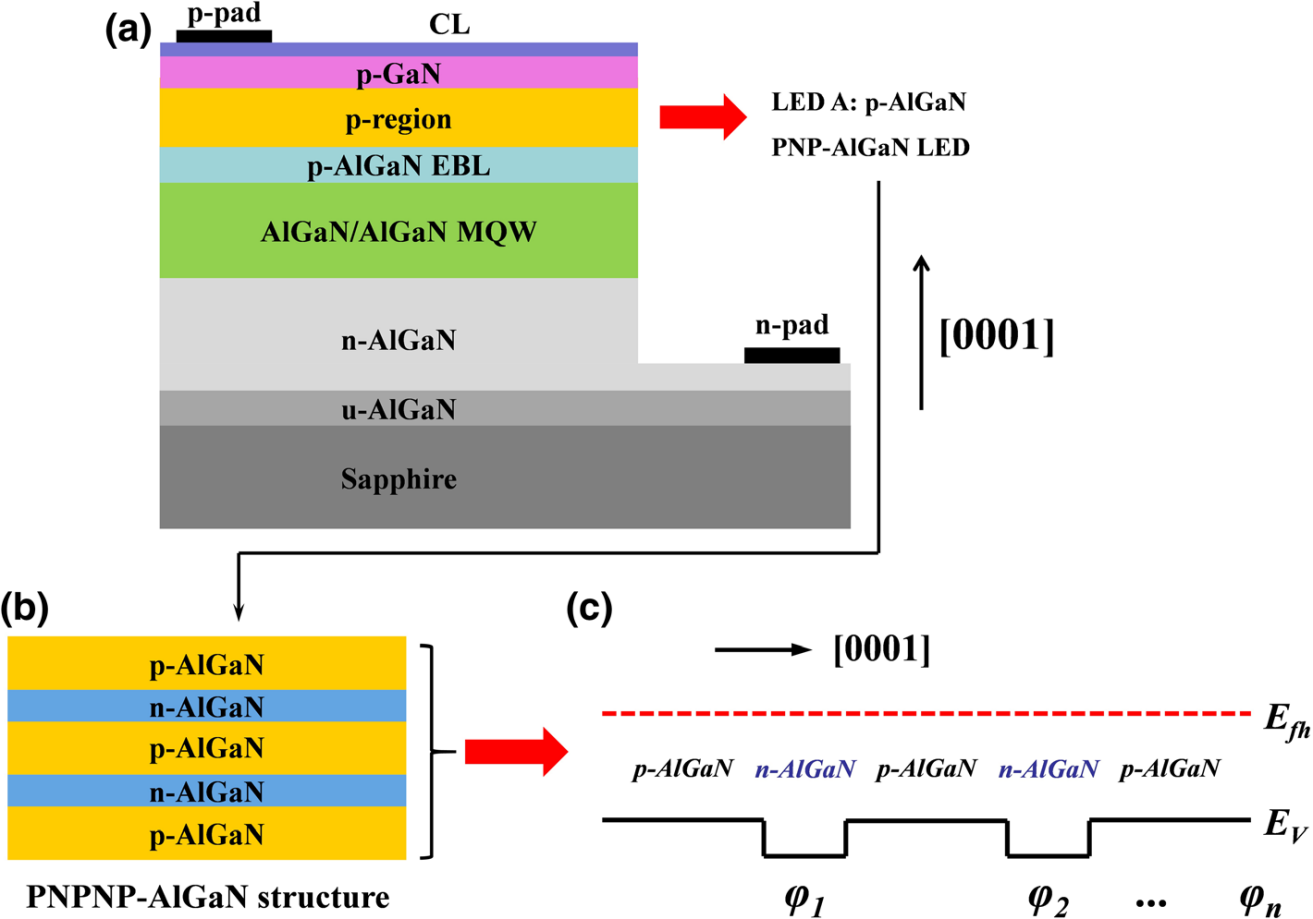
**a** Schematic diagrams for the studied devices (reference LED A and PNPNP-AlGaN LED), **b** schematic diagrams for the PNP-AlGaN structure with two PNP-AlGaN junctions, **c** schematic valence band diagram for the PNP-AlGaN structure with multiple PNP-AlGaN junctions, in which *φ*_1_, *φ*_2_, and *φ*_*n*_ denote the barrier height for each PNP-AlGaN junction along the [0001] orientation and *n* represents the PNP-AlGaN junction number

To better clarify the current spreading mechanisms for AlGaN-based DUV LEDs, different DUV LED devices are designed (see Fig. 1a). All DUV LEDs consist of a 4-μm-thick n-type Al_0.60_Ga_0.40_N layer with the Si doping concentration of 5 × 10^18^ cm^−3^. Next, five periods of 3-nm Al_0.45_Ga_0.55_N/12 nm Al_0.56_Ga_0.44_N MQWs follow. We then cap the MQWs with a 18-nm-thick p-type Al_0.60_Ga_0.40_N electron blocking layer (p-EBL), on which a 198-nm-thick p-type Al_0.40_Ga_0.60_N layer and a 50-nm-thick p-type GaN cap layer are employed as the hole injection layer. The hole concentration for the p-type layers is set to 3 × 10^17^ cm^−3^. For the DUV LEDs with the proposed PNP-AlGaN structures, the conventional p-type bulk Al_0.40_Ga_0.60_N layer is replaced by p-Al_0.40_Ga_0.60_N/n-Al_*x*_Ga_1-*x*_N/p-Al_0.40_Ga_0.60_N layer. Figure 1b presents the schematic structure diagram for the PNP-AlGaN layer. Figure 1c shows the schematic valence band diagram for the PNP-AlGaN structure, from which we can see the barrier for holes. The barrier is generated because of the depletion of the Si dopants in the thin n-Al_*x*_Ga_1-*x*_N layer [26]. This barrier is very important in determining the current flow path and the device performance for DUV LEDs. Detailed analysis will be given subsequently.

**Fig. 2:**
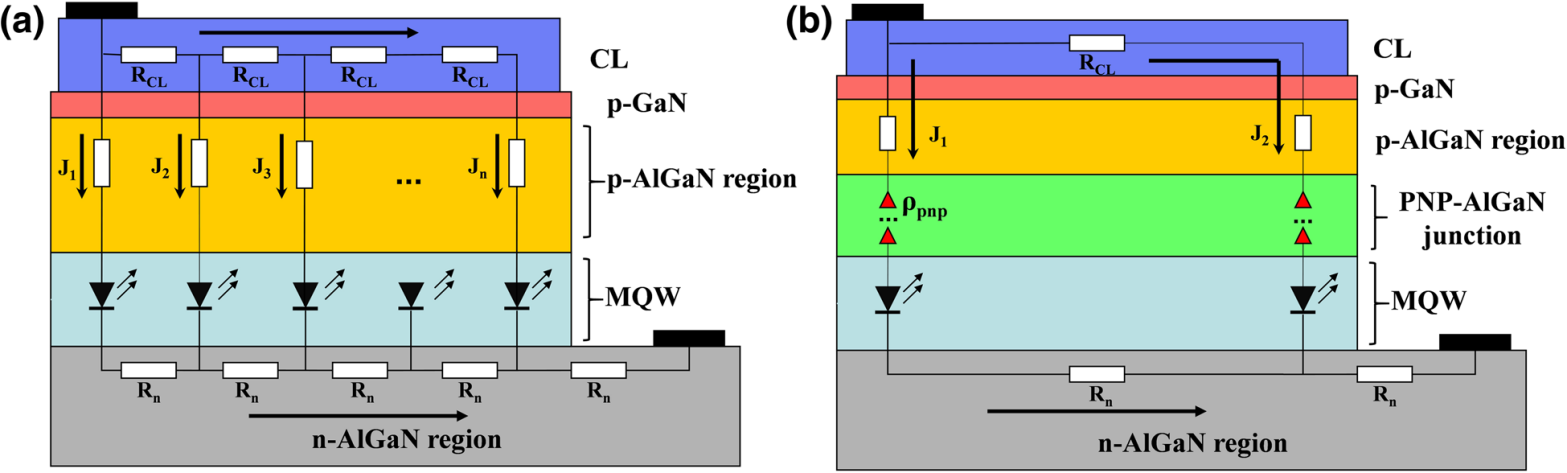
**a** Equivalent circuit of DUV LEDs with lateral current-injection scheme (*J*_1_ > *J*_2_ > *J*_3_ > *J*_4_ > …… > *J*_*n*_) and **b** simplified equivalent circuit of the LED with PNP-AlGaN structure, the current paths (*J*_1_ and *J*_2_) are also shown

To further illustrate the mechanism of the PNP-AlGaN structure in spreading the current, we show the simplified equivalent circuit and the current flow paths for the DUV LED grown on sapphire substrates in Fig. 2a. The current flows both vertically and laterally from the p-AlGaN region to the n-AlGaN region. Normally, the current spreading layer (CL) thickness (i.e., 200 nm for our devices) is much smaller than that of the n-AlGaN layer (i.e., 4 μm for our devices). Hence, the electrical resistance for the CL is much larger than that for the n-AlGaN electron injection layer. Then, the current tends to crowd underneath the p-electrode, i.e., *J*_1_ > *J*_2_ > *J*_3_ > *J*_4_ > ..... > *J*_*n*_, which is known as the current crowding effect [27]. Fortunately, the current crowding effect can be suppressed by incorporating the PNP-AlGaN junction in the p-type hole injection layer, and the underlying mechanism can be interpreted by using Fig. 2b, such that we divide the total current into a vertical part (*J*_*1*_) and a horizontal part (*J*_*2*_). According to Ref. [27], the relationship between *J*_*1*_ and *J*_*2*_ can be linked by Eq. (1) as follows,

1 $$ \frac{J_1}{J_2}\cong \frac{l}{\frac{\rho_p}{\rho_{\mathrm{CL}}}{t}_p+\frac{N\cdot {\rho}_{\mathrm{PNP}}}{\rho_{\mathrm{CL}}}}, $$

where *l* is the length of horizontal current path, *t*_*p*_ is the thickness, *ρ*_*p*_ stands for the vertical resistivity for p-type hole injection layer, *ρ*_CL_ denotes the resistivity of ex situ deposited current spreading layer, *ρ*_PNP_ means the specific interfacial resistivity induced in each PNP-AlGaN junction, and *N* represents the number of the PNP-AlGaN junction. Based on Eq. (1), we infer that we can increase *J*_*2*_ by reducing *ρ*_CL_. Equation (1) also indicates that the proper increase of the vertical resistance (i.e., *ρ*_*p ×*_ *t*_*p*_) also helps to enhance *J*_*2*_. Alternatively, the vertical resistance can become larger by including the *N·ρ*_PNP_. However, the value of *N·ρ*_PNP_ can be affected by the number of PNP-AlGaN junction. Moreover, the value of *ρ*_PNP_ is subject to the doping concentration, the thickness and the AlN composition of the n-Al_*x*_Ga_1-*x*_N layer. Thus, details regarding different PNP-AlGaN junctions will be discussed subsequently.

Investigations into the device physics are conducted by using APSYS [28]. The energy band offset ratio between the conduction band offset and the valence band offset for the AlGaN/AlGaN heterojunction is set to 50:50 [29]. The polarization level is set to 40% for calculating the polarization induced charges at the lattice-mismatched interfaces [30, 31]. The Auger recombination coefficient and the Shockley-Read-Hall (SRH) recombination lifetime are set to be 1.0 × 10^−30^ cm^6^/s [27] and 10 ns [32], respectively. The light extraction efficiency is set to ~ 8% for DUV LEDs [33]. Other parameters on nitrogen-containing III–V semiconductors can be found elsewhere [34].

## Results and Discussions

### Proof of the Effectiveness of the PNP-AlGaN Junction in Spreading the Current for DUV LEDs

**Fig. 3:**
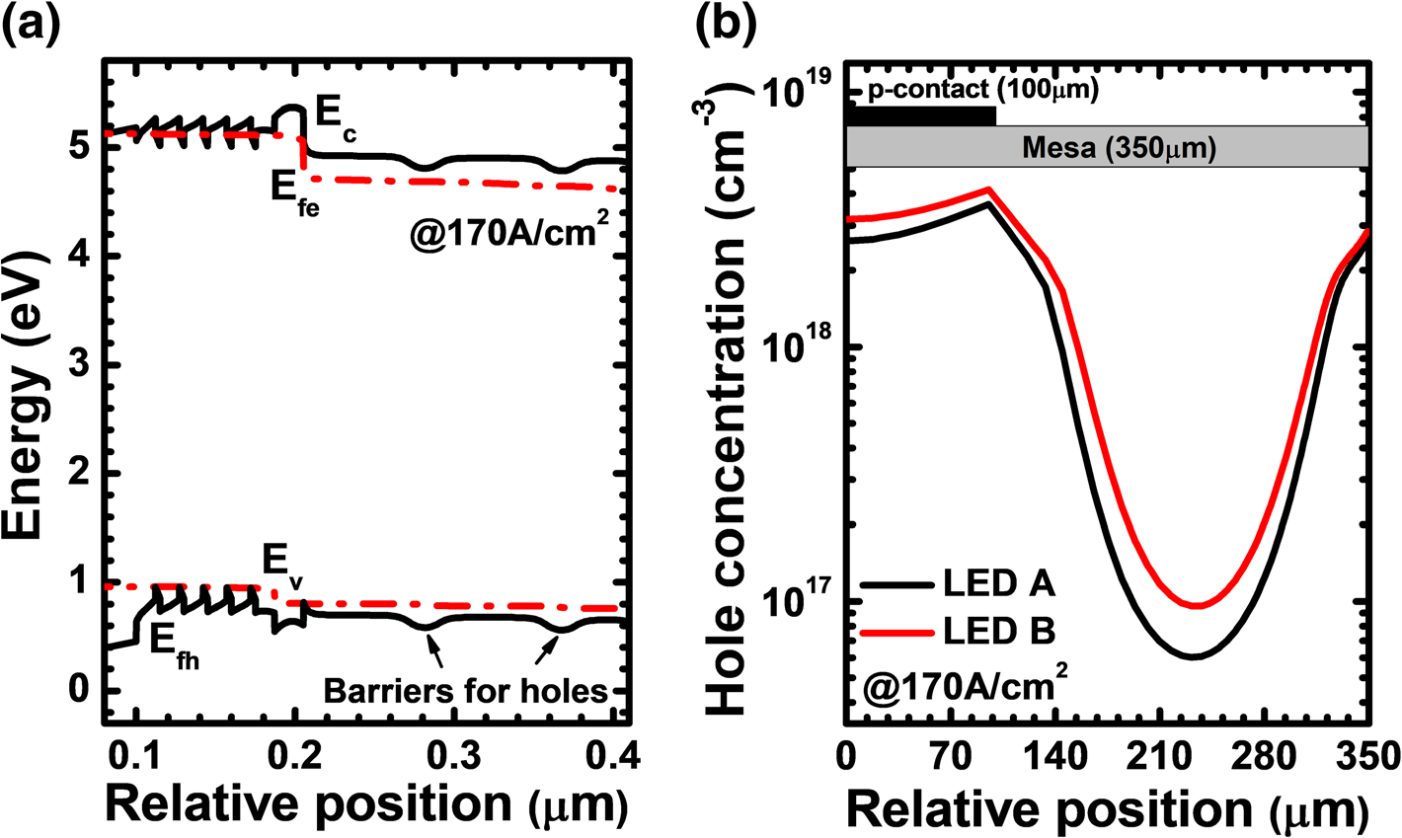
**a** Energy band diagram for LED B at the current density of 170 A/cm^2^. *E*_*c*_, *E*_*v*_, *E*_fe_, and *E*_fh_ denote the conduction band, the valance band, and quasi-Fermi levels for electrons and holes, respectively, **b** lateral hole distribution in the last quantum well for LEDs A and B at the current density of 170 A/cm^2^, respectively

To show the effectiveness of the PNP-AlGaN structure in spreading the current for DUV LED, the reference DUV LED (i.e., LED A) without PNP-AlGaN structure and the DUV LED with the PNP-AlGaN structure (i.e., LED B) are studied. Note the architectural information for the DUV LEDs has been given in section of Research Methods and Physics Models except the PNP-AlGaN configuration for LED B. LED B has two PNP-AlGaN loops, i.e., PNPNP-AlGaN structure. Each PNP-AlGaN junction comprises the p-Al_0.40_Ga_0.60_N/n-Al_0.40_Ga_0.60_N/p-Al_0.40_Ga_0.60_N structure, for which the Si doping concentration in the 20-nm-thick n-Al_0.40_Ga_0.60_N insertion layer is 5.3 × 10^17^ cm^−3^. We calculate and show the energy band diagram for LED B at the current density of 170 A/cm^2^ in Fig. 3a. We can see that, when compared to LED A (energy band are not shown here), the holes will encounter two barriers before being injected into the MQWs. The barriers in the valence band here can effectively spread the current and better homogenize the holes laterally. To further address our point and for the purpose of the demonstration, we show the lateral hole concentration profile in the quantum well closest to the p-EBL [i.e., the last quantum well (LQW)] in Fig. 3b, which finds that the hole distribution in LED B indeed shows a more uniform profile in the LQW. The observations in Fig. 2b agree well with the energy band diagrams in Fig. 3a and our analysis previously, such that the PNP-AlGaN structure proves useful in improving the current spreading effect.

**Fig. 4:**
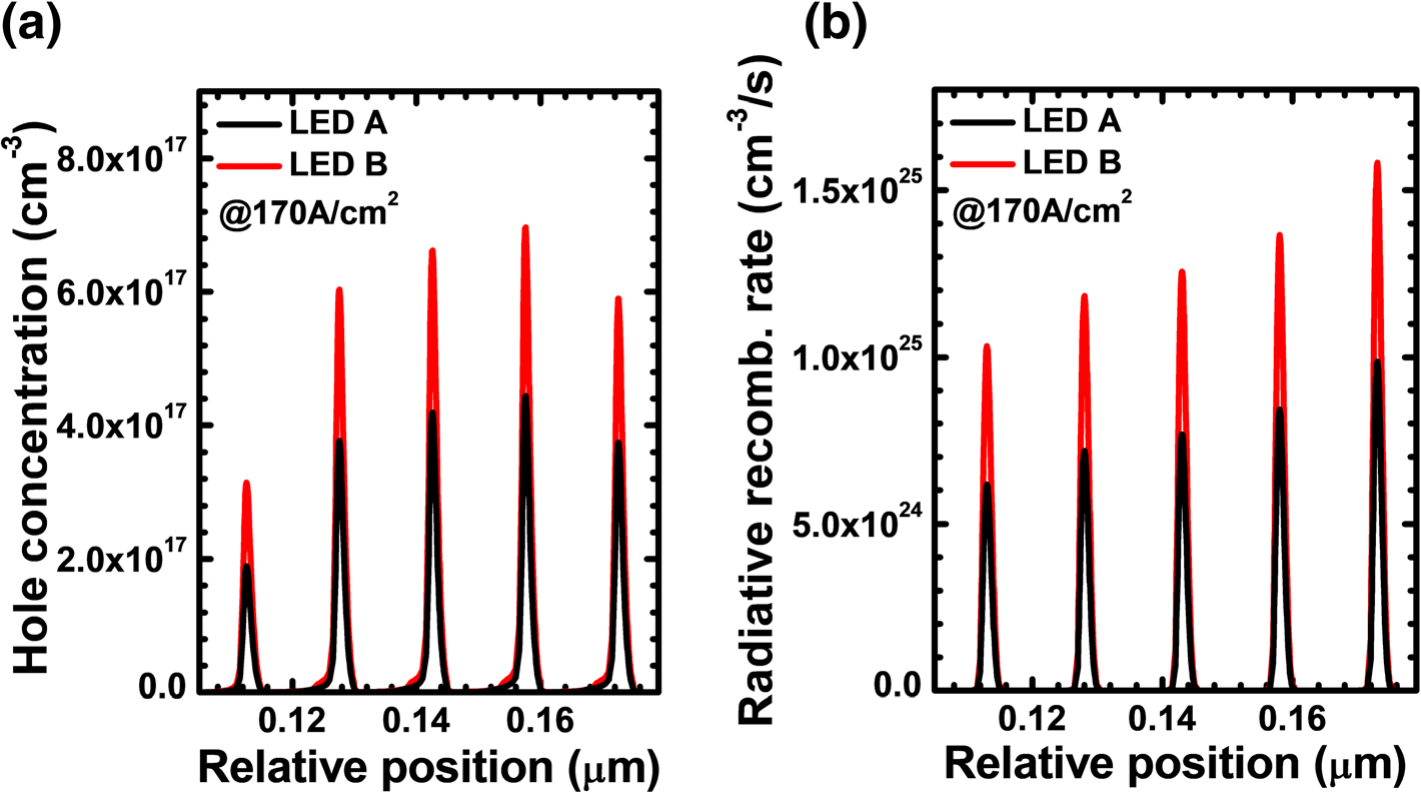
**a** Hole concentration profiles and **b** radiative recombination rate in the MQW region for LEDs A and B at the current density of 170 A/cm^2^, respectively

Next, we show the profiles for the hole concentration and the radiative recombination rate in the MQW region for LEDs A and B in Fig. 4a, b, respectively. Note that to monitor the current spreading effect, the data in Fig. 4a, b are collected at the position of 230 μm apart from the left mesa edge. It is found that the improved current spreading for LED B also enables the promoted hole injection into the MQWs. The improvement of the hole concentration in the MQWs generates the enhanced radiative recombination rate for LED B according to Fig. 4b.

**Fig. 5:**
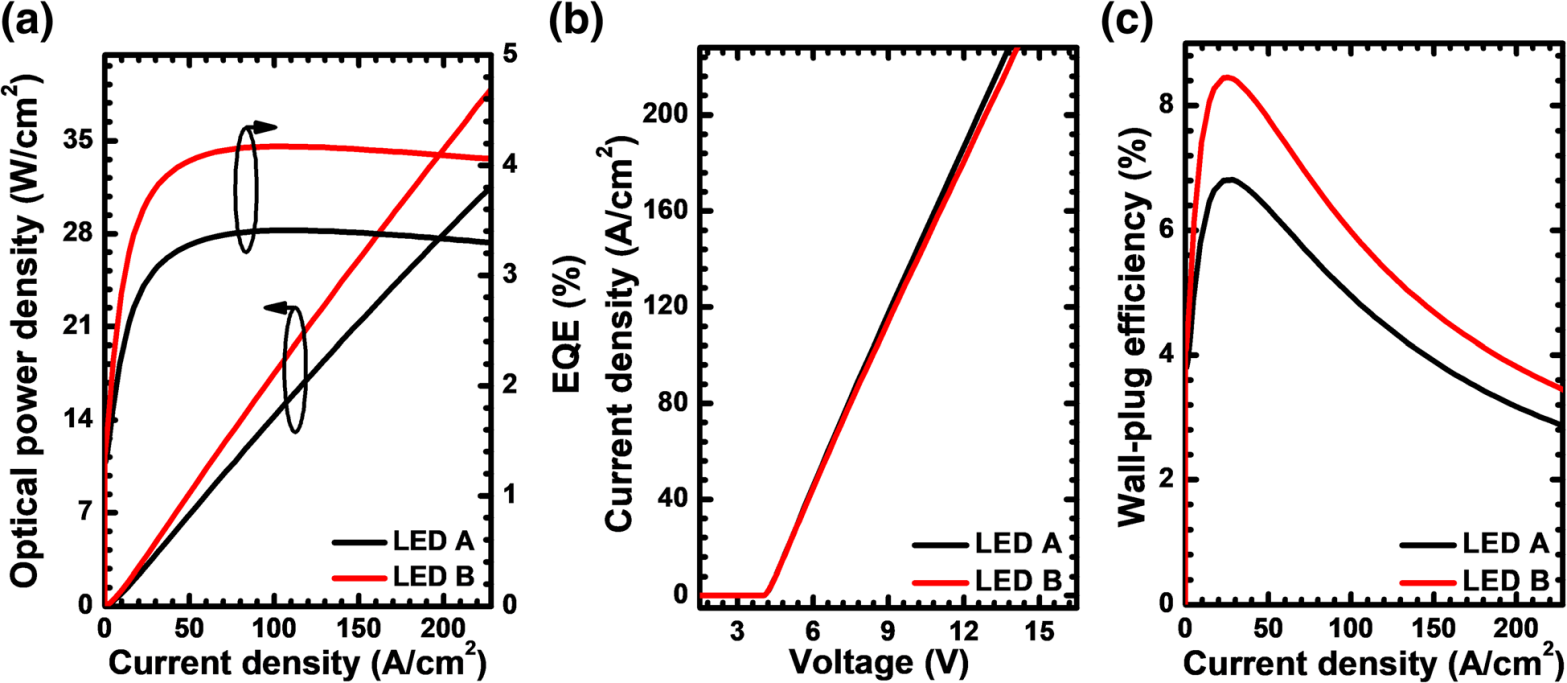
**a** Optical output power density and EQE as a function of the injection current, **b** current-voltage characteristic, **c** WPE in terms of the injection current for LEDs A and B, respectively

Figure 5a then demonstrates the EQE and the optical power density in terms of the injection current density level for LEDs A and B. The EQE levels for LEDs A and B are 3.38% and 4.13%, respectively, showing a performance enhancement of 22.2% at the current density of 170 A/cm^2^. These observed improvements are attributed to the better current spreading effect and the enhanced hole injection into the MQW region for LED B. As has been mentioned previously, the adoption of the PNP-AlGaN structure can lead to the energy barrier in the valence band, which may influence the forward voltage. The speculation is proven when referring to Fig. 5b that demonstrates the slightly increased forward voltage for LED B. Despite the higher forward voltage for LED B, the wall-plug efficiency for LED is still larger than that for LED A according to Fig. 5c, such that the numbers are 3.56% and 4.27% for LEDs A and B at the current density level of 170 A/cm^2^, respectively. If we further compare Fig. 5a, c, we can find that the WPE has a more pronounced droop for LED B, and this is ascribed to the additional voltage drop at the PNP-AlGaN junction. Therefore, it is essentially important to conduct a more comprehensive study revealing the sensitivity of the EQE, forward voltage, and the WPE to different PNP-AlGaN designs.

### Impact of the Thickness for the n-AlGaN Layer on the Device Performance

**Fig. 6:**
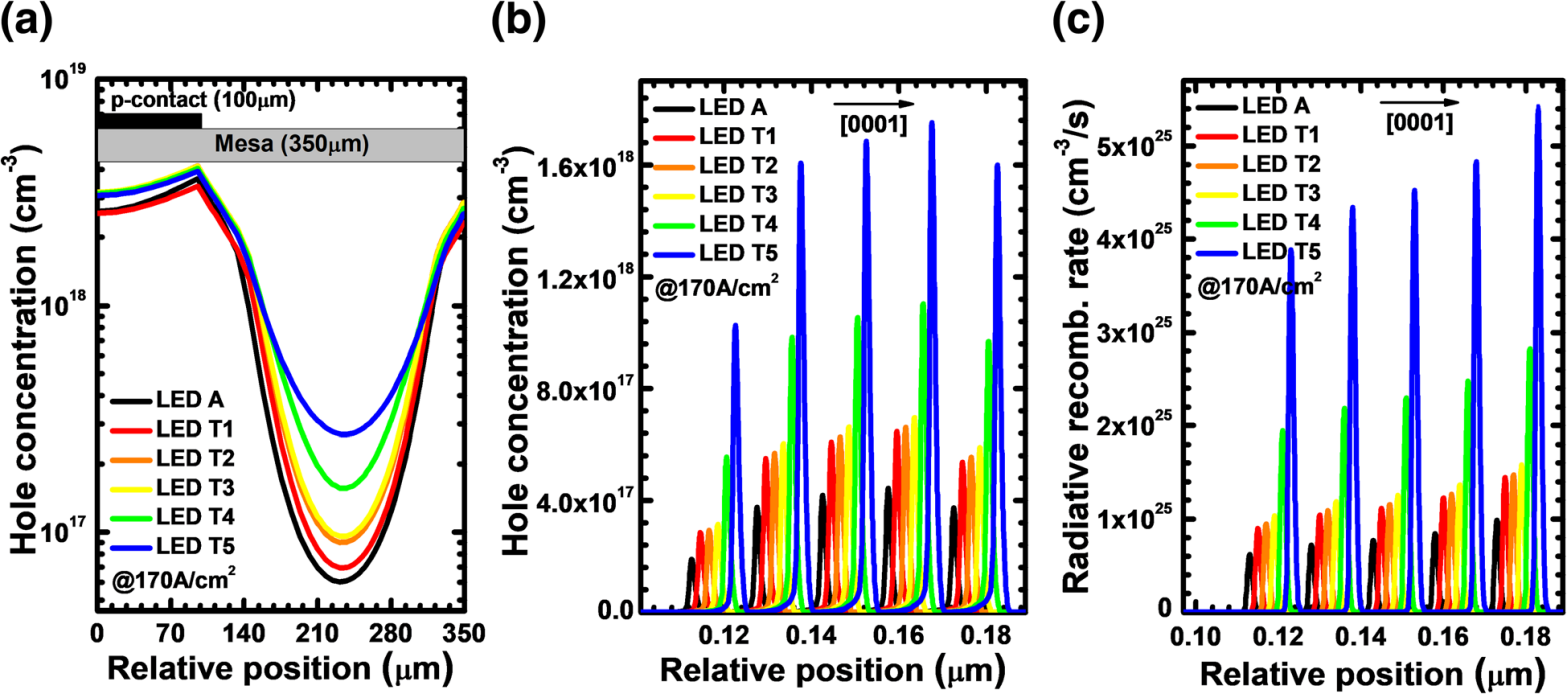
**a** Lateral hole distribution in the last quantum well, **b** hole concentration profiles and **c** radiative recombination rate profiles in the MQW region for LEDs A, T1, T2, T3, T4, and T5 at the current density of 170 A/cm^2^. The plotted curves for panels **b** and **c** are purposely shifted by 2 nm for better resolution

According to Eq. (1), we can conclude that an enhanced horizontal current flow can be obtained by increasing the value of *N·ρ*_PNP_. The barrier height in the PNP-AlGaN junction increases when the n-Al_0.40_Ga_0.60_N layer becomes thick so that a larger *ρ*_PNP_ can be obtained, which is beneficial for the improved current spreading effect. However, once the n-Al_0.40_Ga_0.60_N layer is too thick, more holes in the p-Al_0.40_Ga_0.60_N layer may be depleted, which may sacrifice the electrical conductivity. Therefore, to better illustrate the relationship between the thickness of n-Al_0.40_Ga_0.60_N layer and performance for DUV LEDs, it is necessary to investigate the impact of the n-Al_0.40_Ga_0.60_N layer thickness for the PNP-AlGaN junction on the current spreading, the hole injection, the EQE, the forward voltage, and the WPE. For that purpose, we vary the values of the n-Al_0.40_Ga_0.60_N layer thickness among 6, 13, 20, 27, and 34 nm, and the devices are called LEDs T1, T2, T3, T4, and T5, respectively. Table 1 summarizes the valence band barrier height for each PNP-AlGaN junction, which shows that the barrier height increases as the n-Al_0.40_Ga_0.60_N layer thickness increases, proving that the increase of the n-Al_0.40_Ga_0.60_N layer thickness enables the large *N·ρ*_*PNP*_, thus increasing the horizontal current *J*_*2*_. Figure 6a then shows the lateral hole concentration profiles in the last quantum well for LED A without the PNP-AlGaN structured current spreading layer and the LEDs with various n-Al_0.40_Ga_0.60_N layer thicknesses at the current density of 170 A/cm^2^. It can be seen apparently that the holes become more evenly distributed in the last quantum well as the thickness for the n-Al_0.40_Ga_0.60_N insertion layer increases.

**Table 1:** Valence band barrier height for each PNP-AlGaN junction of LEDs T1, T2, T3, T4, and T5

LEDs	T1	T2	T3	T4	T5
*φ*_1_ (eV)	0.129	0.156	0.208	0.254	0.273
*φ*_2_ (eV)	0.129	0.156	0.210	0.256	0.274

Then, we show the hole concentration profiles and radiative recombination rate profiles in the MQW region for all studied devices at the current density of 170 A/cm^2^ in Fig. 6b, c, respectively. The hole concentration and radiative rate profiles are collected at the position of 230 μm apart from the left-hand mesa edge. For the better visual resolution, the hole concentration and radiative recombination rate profiles for LEDs A, T1, T2, T3, T4, and T5 are spatially shifted by 2 nm in Fig. 6b, c, respectively. It is clearly shown that LED A has the lowest hole concentration and thus the lowest radiative recombination rate in the MQW region. The hole concentration and radiative recombination rate in the MQW region increase with the increasing thickness of the n-Al_0.40_Ga_0.60_N layer.

**Fig. 7:**
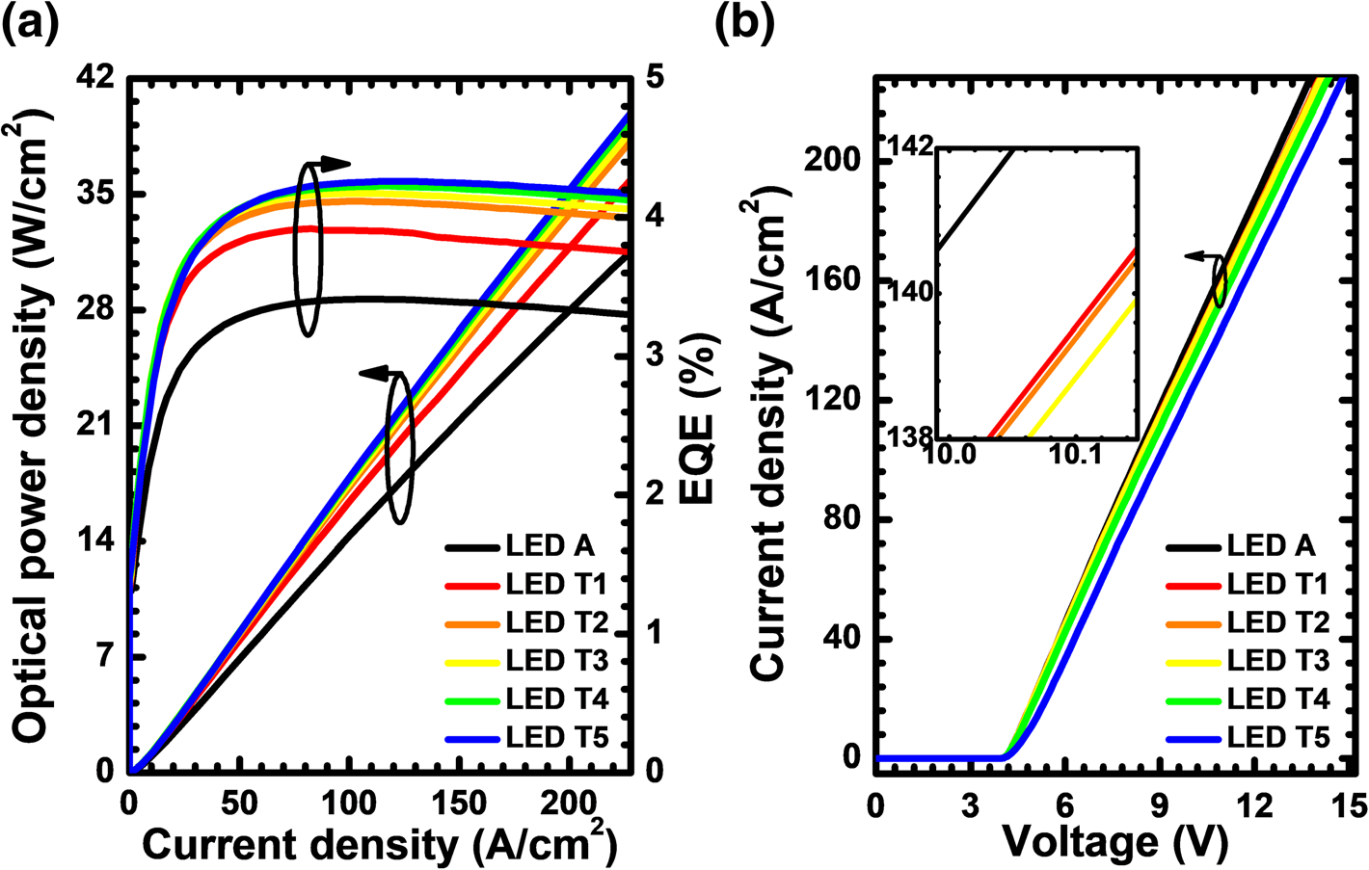
**a** Optical output power density, **b** current-voltage characteristics for LEDs A, T1, T2, T3, T4, and T5. Inset figure shows the zoom-in current-voltage curves

**Fig. 8:**
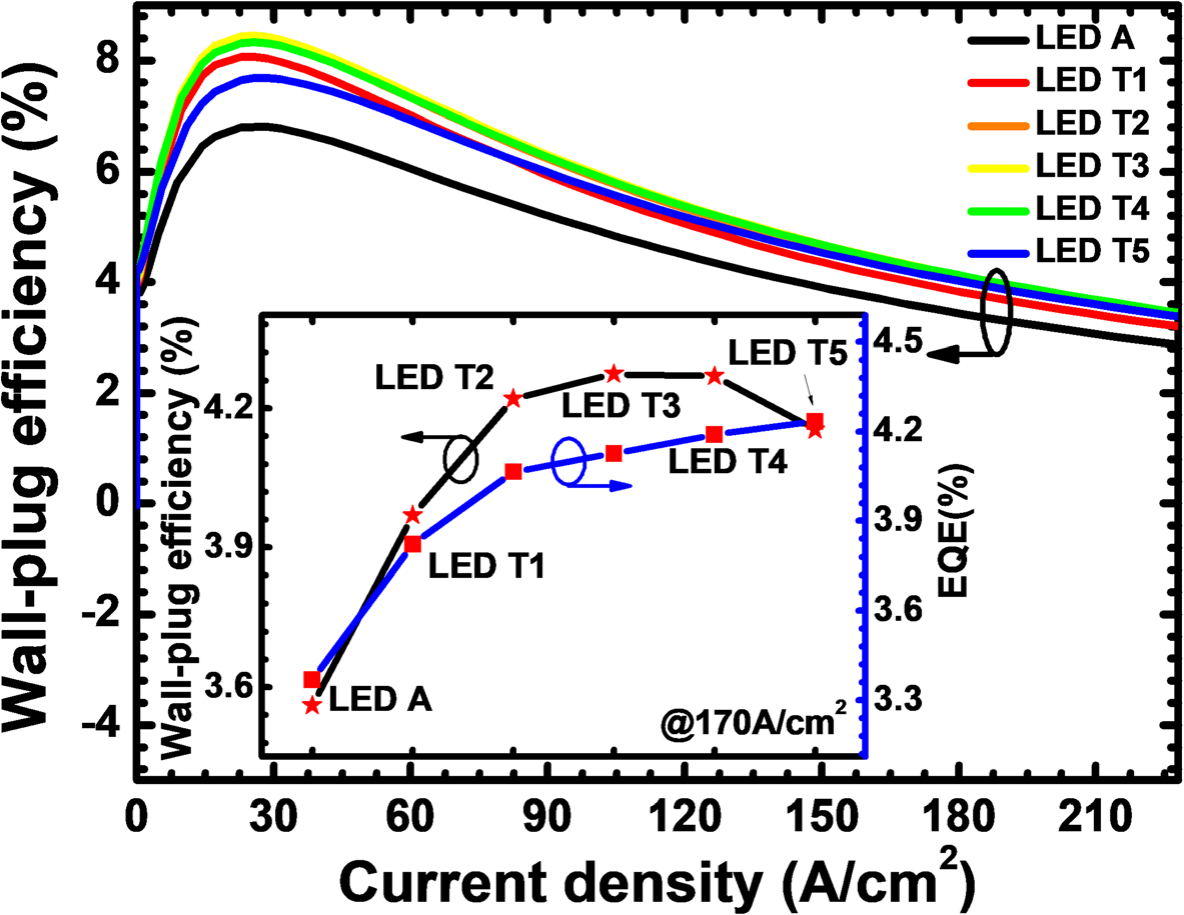
WPE as a function of the injection current for LEDs A, T1, T2, T3, T4, and T5. Inset figure shows the WPE and EQE for the studied LEDs with various thicknesses of the n-Al_0.40_Ga_0.60_N layer for the PNP-AlGaN junction at the current density of 170 A/cm^2^

The observed results shown in Fig. 6c agree well with the EQE, and the optical power density that are presented in Fig. 7a, such that the increasing thickness of the n-Al_0.40_Ga_0.60_N layer in the PNP-AlGaN junction can improve the EQE and the optical power density. However, the valence band barrier height for holes in each PNP-AlGaN junction becomes large once the n-Al_0.40_Ga_0.60_N layer is thickened according to Table 1, which correspondingly increases the forward voltage for the proposed DUV LEDs as shown in Fig. 7b. Therefore, the impact of the n-Al_0.40_Ga_0.60_N layer thickness for the PNP-AlGaN current spreading on the LED performance shall be evaluated by demonstrating the relationship between the WPE and the injection current density (see Fig. 8). We can see that the WPE does not monotonically increases with the increasing n-Al_0.40_Ga_0.60_N layer thickness. The EQE and the WPE in terms of the n-Al_0.40_Ga_0.60_N layer thickness are illustrated in the inset of Fig. 8. For the proposed device architectures in this work, the WPE reaches the highest value when the n-Al_0.40_Ga_0.60_N insertion layer is 20 nm thick and it decreases as the n-Al_0.40_Ga_0.60_N insertion layer becomes thicker. We attribute this phenomenon to the increased vertical resistance when the n-Al_0.40_Ga_0.60_N layer thickness getting thicker, and this consumes more electrical power. Therefore, the n-AlGaN insertion layer thickness for the PNP-AlGaN junction shall be carefully optimized. In this section, we set the AlN composition of 40%, i.e., n-Al_0.40_Ga_0.60_N for the purpose of demonstration, and we believe the optimized thickness for the n-AlGaN insertion layer shall become smaller if one increases the AlN composition.

### Impact of the Doping Concentration of the n-AlGaN Layer on the Device Performance

Besides the n-AlGaN layer thickness, the doping concentration for the n-AlGaN layer can also modify the valence band barrier height for holes, thus affecting *N·ρ*_PNP_. To more accurately study the impact of the doping concentration for the n-AlGaN layer on the current spreading effect and the optical performance for DUV LEDs with the PNP-AlGaN junctions, we set the doping concentration of 1.3 × 10^17^, 5.3 × 10^17^, 9.3 × 10^17^, 1.33 × 10^18^, and 1.73 × 10^18^ cm^−3^ of the n-AlGaN layers for LEDs D1, D2, D3, D4, and D5, respectively. The thickness for the n-AlGaN layer is set to 20 nm, and two PNP-AlGaN junctions are adopted. The AlN composition is 40%, i.e., n-Al_0.40_Ga_0.60_N.

**Fig. 9:**
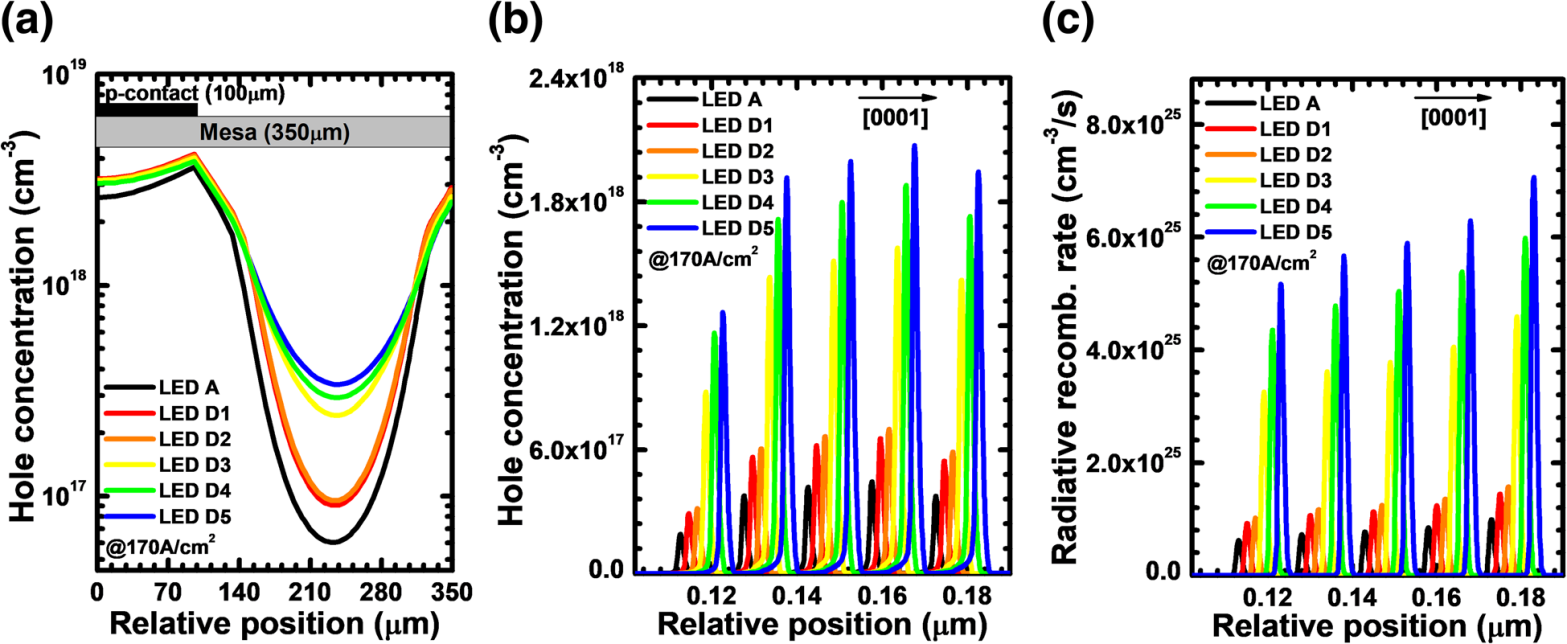
**a** Lateral hole distribution in the last quantum well, **b** hole concentration profiles, and **c** radiative recombination rate profiles in the MQW region or LEDs A, D1, D2, D3, D4, and D5 at the current density of 170 A/cm^2^. The plotted curves for panels **b** and **c** are purposely shifted by 2 nm for better resolution

Table 2 shows that the valence band barrier height for holes increases when the Si doping concentration for the n-Al_0.40_Ga_0.60_N layer of the PNP-AlGaN junction becomes high. The increased valence band barrier height indicates the large *N·ρ*_PNP_, which simultaneously yields the high horizontal current of *J*_*2*_. According to Eq. (1), the increased current spreading is accompanied by the more uniform lateral hole concentration profile, and therefore, we show, in Fig. 9a, that the lateral hole distribution in the last quantum well turns to be more homogenized once the PNP-AlGaN junction is doped for DUV LEDs when compared to LED A. Furthermore, the lateral holes become more evenly distributed once the Si doping concentration for the n-Al_0.40_Ga_0.60_N layer of the PNP-AlGaN junction increases.

**Table 2:** Valence band barrier height for PNP-AlGaN junction of LEDs D1, D2, D3, D4, and D5

LEDs	D1	D2	D3	D4	D5
*φ*_1_ (eV)	0.150	0.208	0.271	0.285	0.292
*φ*_2_ (eV)	0.151	0.210	0.272	0.285	0.292

Then, we show the hole concentration profiles and radiative recombination rate profiles in the MQW region for all studied devices at the current density of 170 A/cm^2^ in Fig. 9b, c, respectively, which are collected at the position of 230 μm apart from the left mesa edge. It is clearly shown that LED A has the lowest hole concentration and the poorest radiative recombination rate in the MQW region. The hole concentration and radiative recombination rate in the MQW region increase with the increasing doping concentrations of the n-Al_0.40_Ga_0.60_N layers for the LEDs with PNP-AlGaN junctions. The enhanced hole concentration level in the MQW for LEDs D1, D2, D2, D3, D4, and D5 is ascribed to the better current spreading effect, thanks to the PNP-AlGaN junction.

**Fig. 10:**
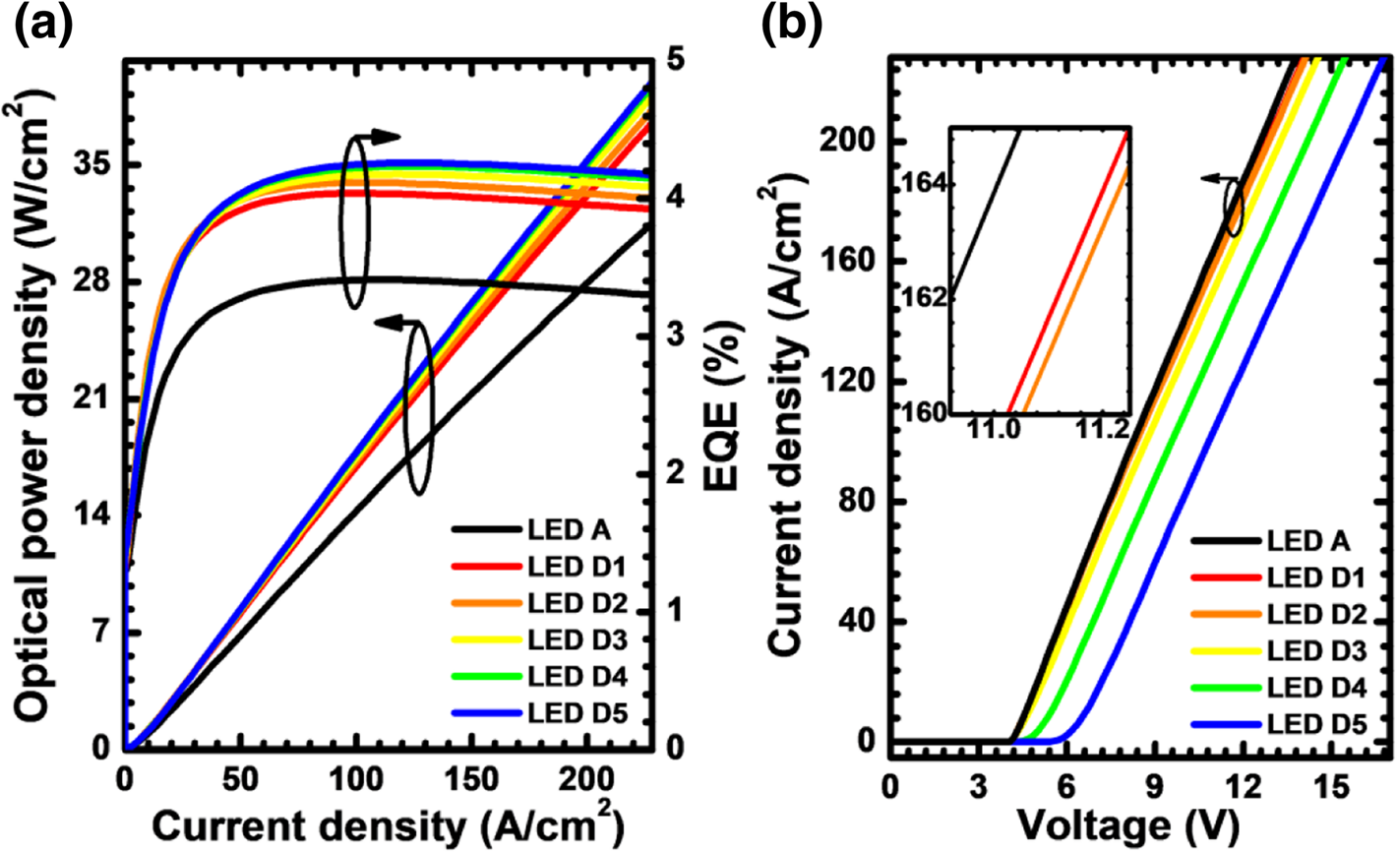
**a** Optical output power density and EQE as a function of the injection current, **b** current-voltage characteristics for LEDs A, D1, D2, D3, D4, and D5. Inset figure shows the zoom-in current-voltage curves

**Fig. 11:**
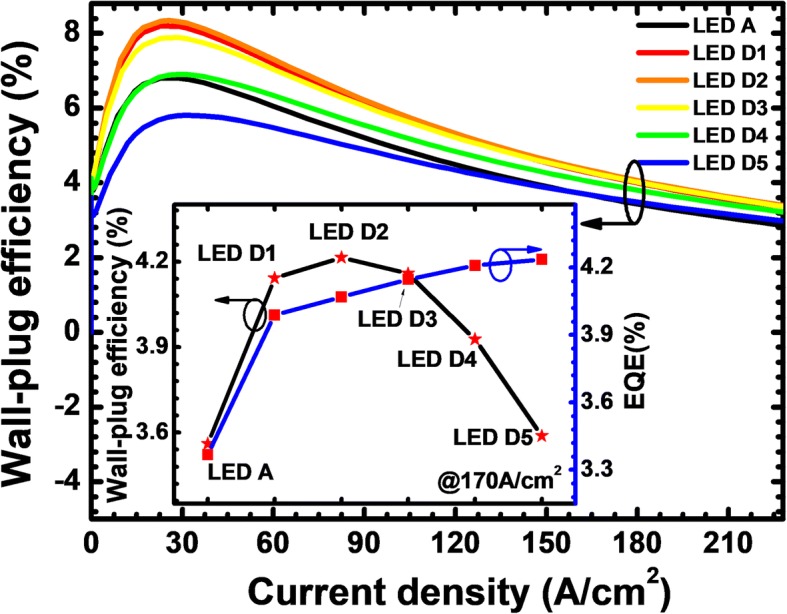
WPE as a function of the injection current for LEDs A, D1, D2, D3, D4, and D5. Inset figure shows the WPE and EQE for the studied LEDs with various doping concentrations of the n-Al_0.40_Ga_0.60_N layer at the current density of 170 A/cm^2^

We then further calculate and present the EQE and the optical power density in terms of the injection current density for the investigated devices in Fig. 10a. The observed EQE is consistent with the results in Fig. 9b, c, such that the EQE can be improved once the PNP-AlGaN junction is employed. More than that, as the Si doping concentration in the n-Al_0.40_Ga_0.60_N layer for the PNP-AlGaN junction increases, the EQE can be further promoted, thanks to the better current spreading. Figure 10b compares the forward operating voltage for the investigated devices. It is shown that the forward operating voltage increases with the increasing of doping concentration in the n-Al_0.40_Ga_0.60_N layer. Note that as the Si doping concentrations are 1.33 × 10^18^ and 1.73 × 10^18^ cm^−3^, the turn-on voltage shows a significant increase, which indicates that the PNP-AlGaN built-in junction behaves a parasitic diode when the Si doping in the n-Al_0.40_Ga_0.60_N layer increases to a very high level. To more accurately assess the performance of the DUV LEDs with different PNP-AlGaN junctions, Fig. 11 exhibits WPE as a function of the injection current density for LED A, D1, D2, D3, D4, and D5. Clearly, we can see that the WPE is the lowest for LED D5, which is because of the largest forward voltage consumption. The inset for Fig. 11 also indicates that the WPE is more sensitive to the Si doping concentration of the n-Al_0.40_Ga_0.60_N layer than the EQE. It is worth concluding that the high Si doping concentration of the n-Al_0.40_Ga_0.60_N layer can indeed improve the current spreading layer and increase the photon generation rate. Nevertheless, the additional forward voltage drop at the PNP-AlGaN junctions consumes more electrical power, thus limiting the WPE. The findings in this section also illustrate that the Si doping concentration in the n-Al_*x*_Ga_1-*x*_N layer shall be properly reduced if one increases the AlN composition and/or the thickness of the n-Al_*x*_Ga_1-*x*_N layer for the PNP-AlGaN junction, since by doing so, one can obtain both the improved EQE and the decent WPE.

### Impact of PNP-AlGaN Junction Number on the Device Performance

**Fig. 12:**
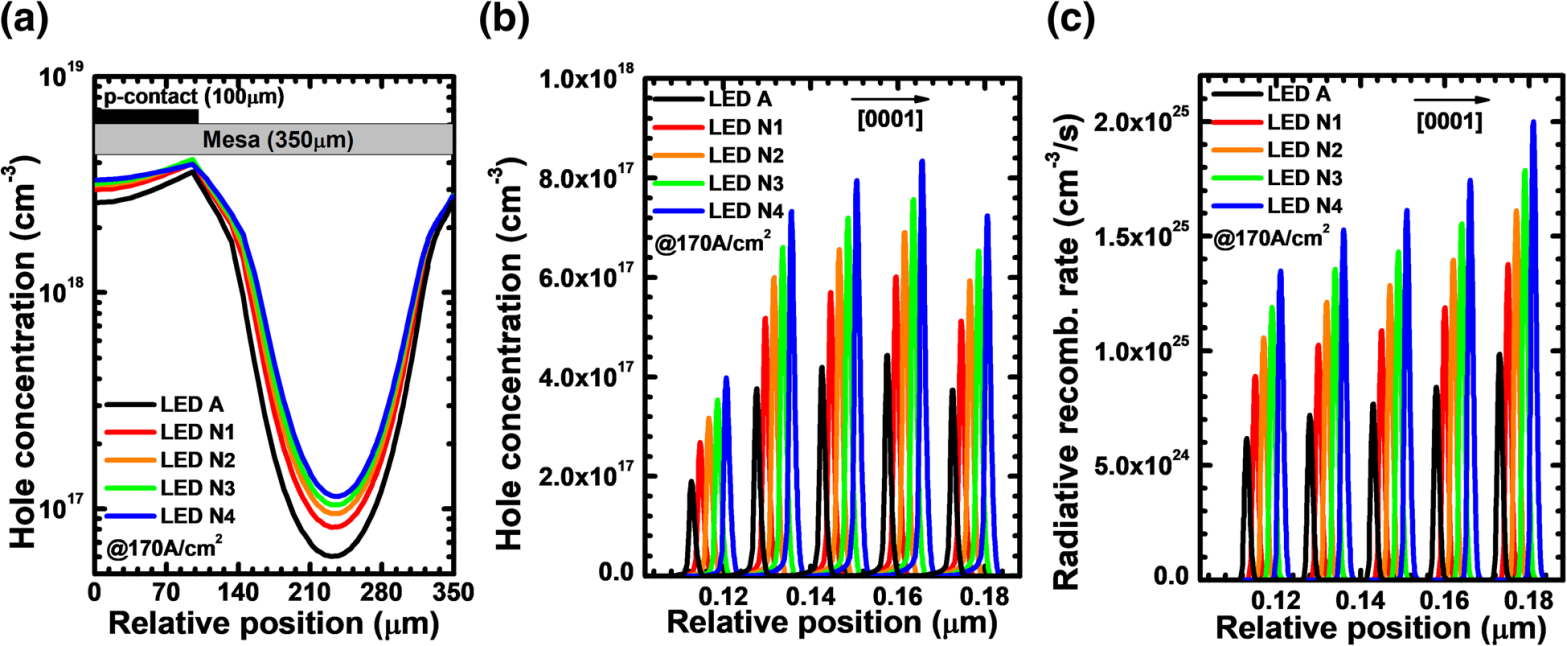
**a** Lateral hole distribution in the last quantum well, **b** hole concentration profiles, and **c** radiative recombination rate profiles in the MQW region for LEDs A, N1, N2, N3, and N4 at the current density of 170 A/cm^2^. The plotted curves for panels **b** and **c** are purposely shifted by 2 nm for better resolution

In this section, the impact of the number of the PNP-AlGaN junction on the electrical and optical performances for DUV LEDs is studied. For the purpose of demonstration, we fix the doping concentration and the thickness of the n-AlGaN layer to 5.3 × 10^17^ cm^−3^ and 20 nm, respectively. The AlN composition is selected to 0.40 such as n-Al_0.40_Ga_0.60_N. We adopt different loops for PNP-AlGaN junction, i.e., the loop numbers are set to 1, 2, 3, and 4 for LEDs N1, N2, N3, and N4, respectively. We firstly calculate and present the valence band barrier height for each PNP-AlGaN junction in Table 3. It can be obviously read that the increase of the PNP-AlGaN junction number makes the overall *N·ρ*_PNP_ high. We then calculate and demonstrate the lateral distribution for the holes in the last quantum well for LEDs A, N1, N2, N3, and N4 at the current density of 170 A/cm^2^ (see Fig. 12a). It shows that the hole distribution in the last quantum well becomes more uniform as more PNP-AlGaN junctions are incorporated. The results in Fig. 12a further support the predictions made by Eq. (1).

**Table 3:** Valence band barrier height for each PNP-AlGaN junction of LEDs N1, N2, N3, and N4

LEDs	N1	N2	N3	N4
*φ*_1_ (eV)	0.208	0.208	0.209	0.210
*φ*_2_ (eV)	–	0.210	0.210	0.210
*φ*_3_ (eV)	–	–	0.213	0.211
*φ*_4_ (eV)	–	–	–	0.236

Then, we show the hole concentration and radiative recombination rate profiles in the MQW region for LEDs A, N1, N2, N3, and N4 at the current density of 170 A/cm^2^ in Fig. 12b, c, respectively. The hole and radiative recombination rate profiles are probed at the position of 230 μm apart from the left mesa edge. It is indicated that the hole concentration and radiative recombination rate increase if the number of the PNP-AlGaN junction is more. It is worth mentioning here that we do not increase the value of *N* beyond 4, since when the *N* is further increased, the thickness of the remaining p-Al_0.40_Ga_0.60_N layer becomes so thin that the holes may be depleted by the ionized Si dopants and the hole supply can be insufficient.

**Fig. 13:**
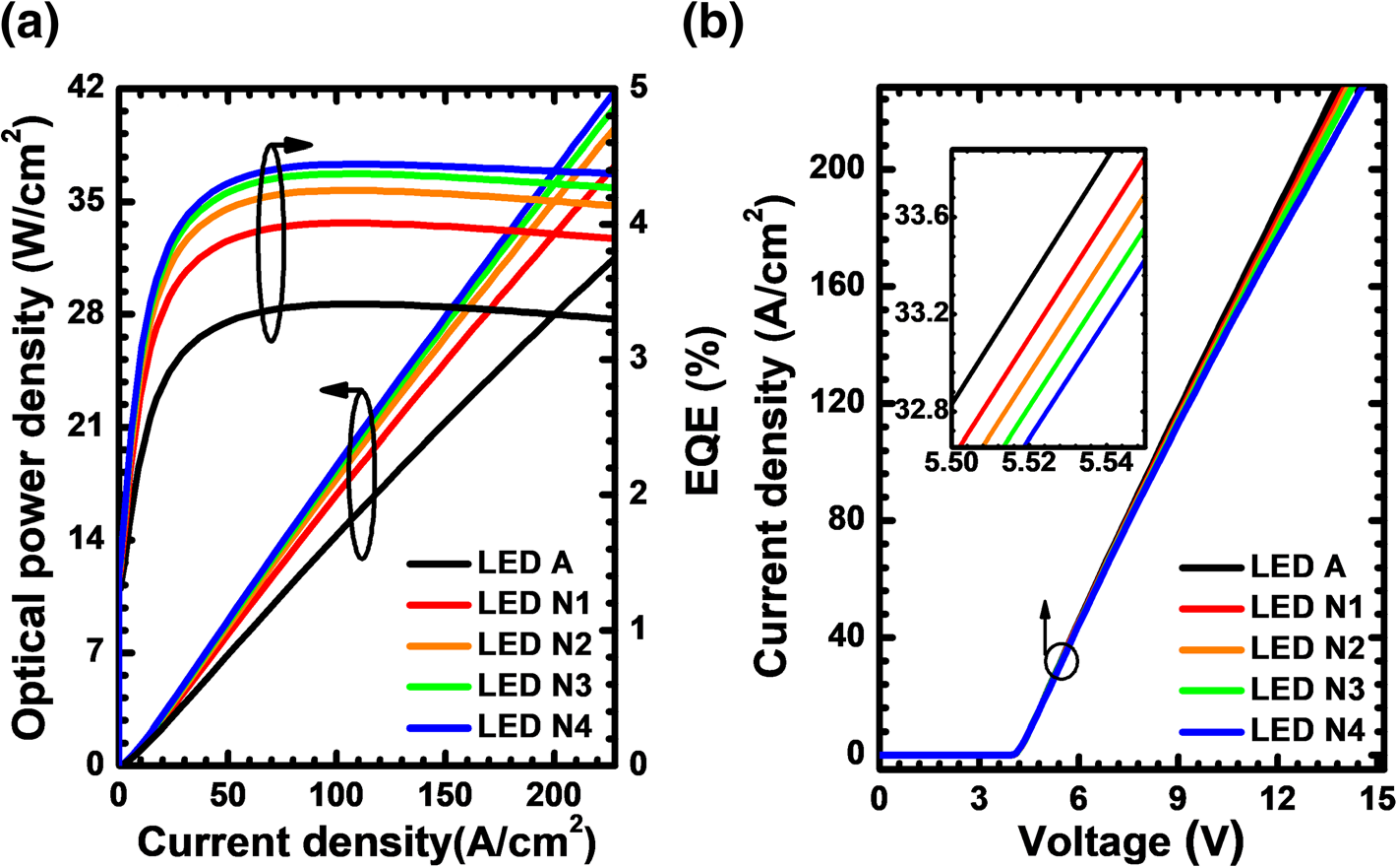
**a** Optical output power density and EQE as a function of the injection current, **b** current-voltage characteristic for LEDs A, N1, N2, N3, and N4. Inset figure shows the zoom-in current-voltage curves

**Fig. 14:**
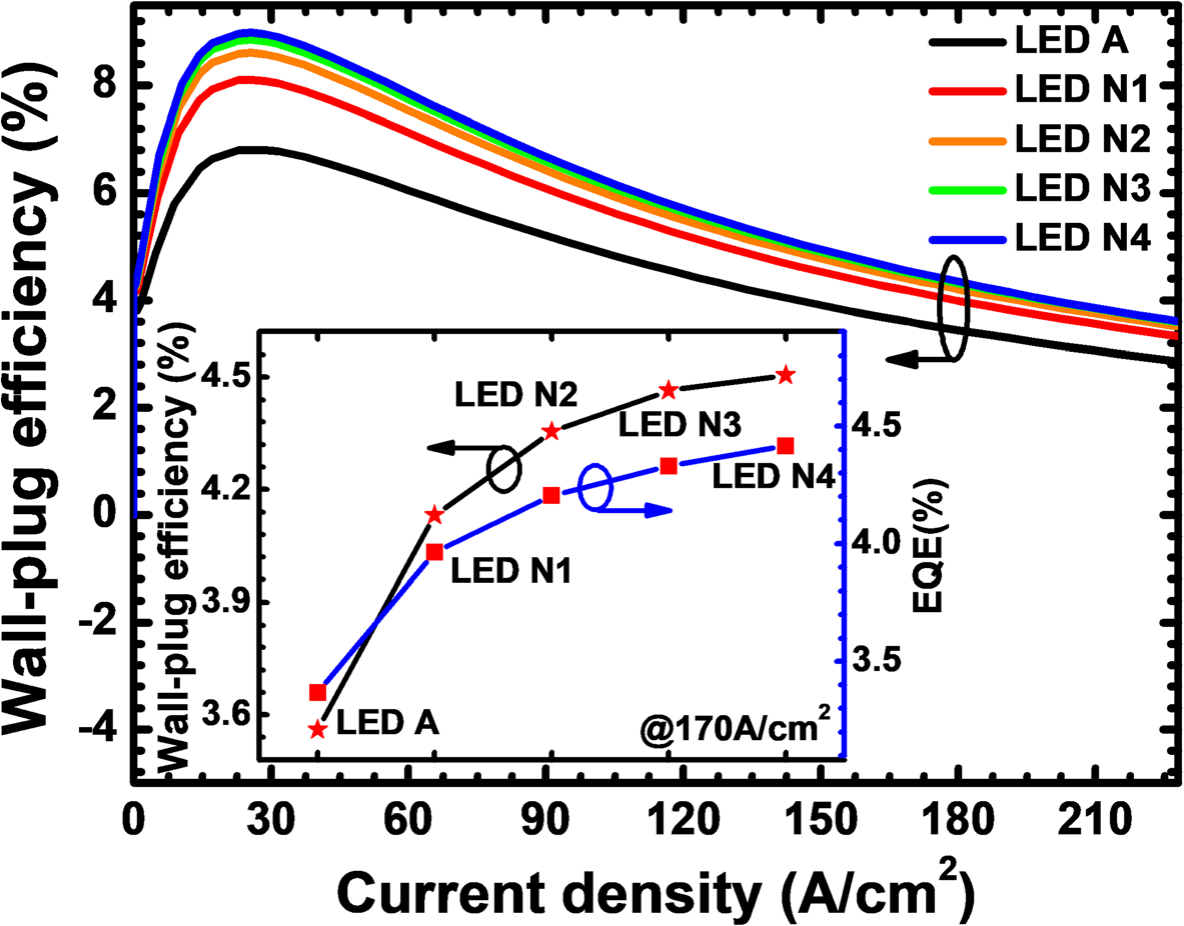
WPE as a function of the injection current for LEDs A, N1, N2, N3, and N4. Inset figure shows the WPE and EQE for LEDs with various number of PNP-AlGaN junction at the current density of 170 A/cm^2^

Thanks to the improved current spreading effect, the enhanced hole concentration in the MQW region, LEDs N1, N2, N3, and N4 consequently promote the EQE and optical power density when compared with LED A (see Fig. 13a). Figure 13b demonstrates that the forward operating voltage for the suggested DUV LEDs also increases if more PNP-AlGaN junctions are incorporated. Fortunately, the increase of the forward voltage for LEDs N1, N2, N3, and N4 does not reduce the WPE according to Fig. 14. Further investigations into the inset of Fig. 14 can illustrate that both the EQE and WPE tend to approach a saturation level as the number of the PNP-AlGaN junction increases. Therefore, we believe that, as has also been pointed out previously, further increase of the number for the PNP-AlGaN junction may deplete the holes and correspondingly degrade the hole supply capability, hence making little contribution in enhancing the EQE and the WPE for the proposed device architectures in this work.

### Impact of the AlN Composition for n-AlGaN Layer on the Device Performance

**Fig. 15:**
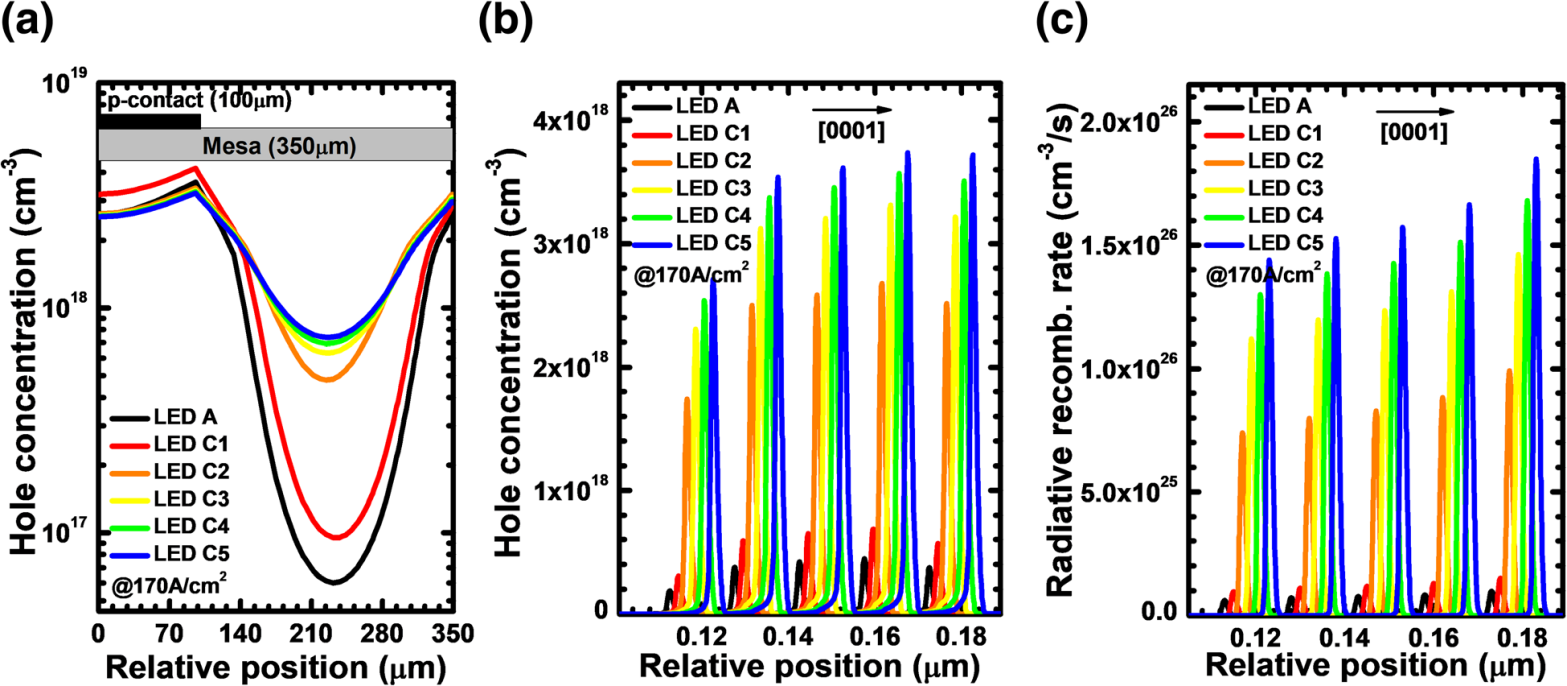
**a** Lateral hole distribution in the last quantum well, **b** hole concentration profiles, and **c** radiative recombination rate profiles in the MQW region for LEDs A, C1, C2, C3, C4, and C5 at the current density of 170 A/cm^2^. The plotted curves for panels **b** and **c** are purposely shifted by 2 nm for better resolution

Lastly, we modify the *ρ*_PNP_ by varying the AlN composition of the n-AlGaN layer for the PNP-AlGaN junction. The values for the AlN composition of the n-AlGaN layer are set to 0.40, 0.43, 0.46, 0.49, and 0.51 for LEDs C1, C2, C3, C4, and C5, respectively. The thickness and the Si doping concentration of the n-AlGaN layer are set to 20 nm and 5.3 × 10^17^ cm^−3^, respectively. We adopt two PNP-AlGaN junctions for LEDs C1, C2, C3, C4, and C5. The AlN composition for the rest p-AlGaN layers is fixed to 0.40. Table 4 demonstrates the valence band barrier height for the PNP-AlGaN junction with different AlN compositions in the n-AlGaN insertion layer. It is easily understandable that the increased AlN composition in the n-AlGaN layer gives rise to the larger valence band barrier height for holes. Figure 15a exhibits the lateral distributions for holes in the last quantum well for LEDs A, C1, C2, C3, C4, and C5 at the current density of 170 A/cm^2^. The current spreading effect is significantly improved as the AlN composition of the n-AlGaN layer increased up to 0.43. It seems that the holes cannot be further soundly spreaded when the AlN composition of the n-AlGaN layer exceeds 0.43 for our structures, because a too much high AlN composition in the n-AlGaN may block the hole injection.

**Table 4:** Valence band barrier height for each PNP-AlGaN junction of LEDs C1, C2, C3, C4, and C5

LEDs	C1	C2	C3	C4	C5
*φ*_1_ (eV)	0.208	0.271	0.290	0.297	0.300
*φ*_2_ (eV)	0.210	0.271	0.290	0.297	0.301

**Fig. 16:**
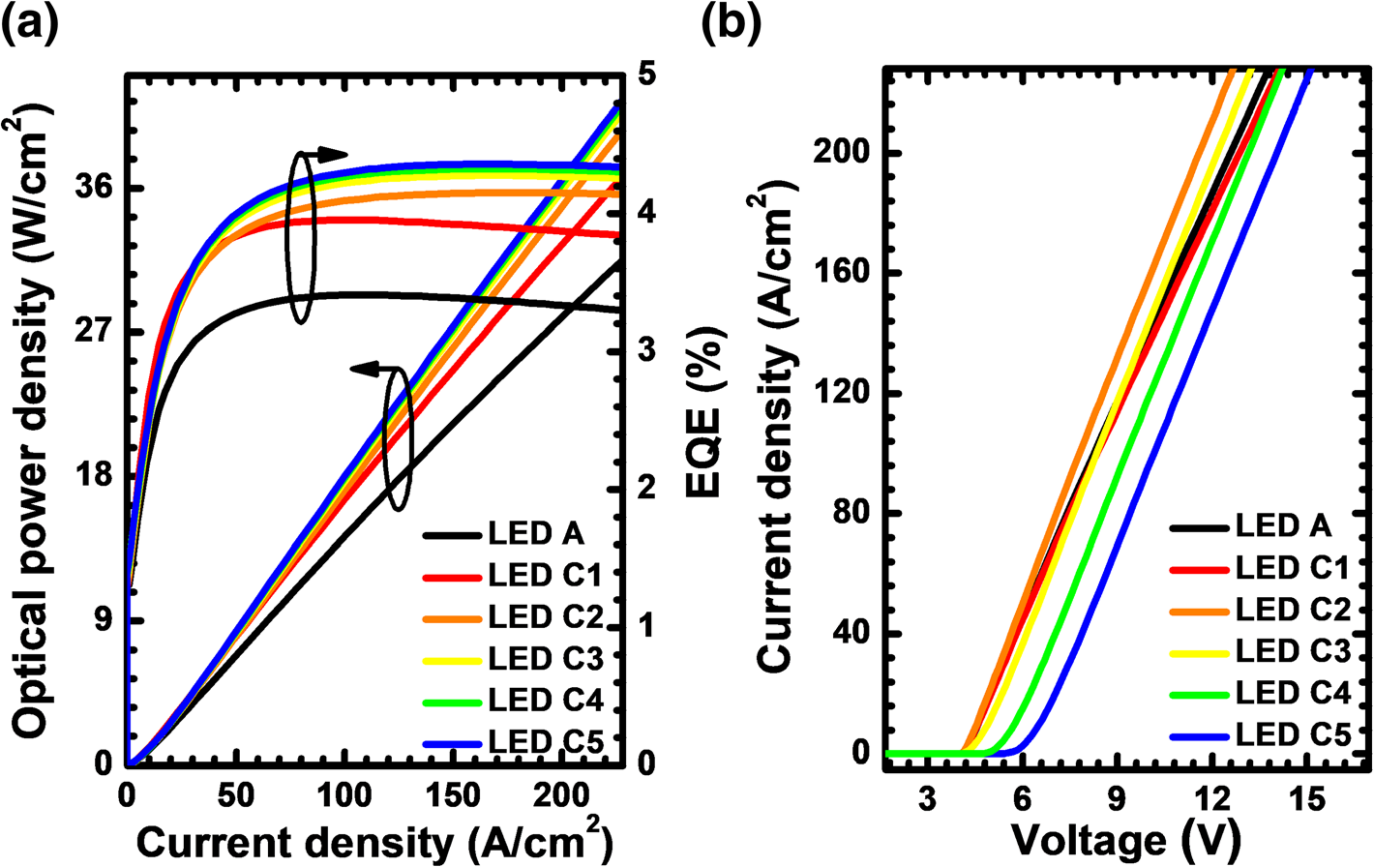
**a** Optical output power density and EQE as a function of the injection current and **b** current-voltage characteristics for LEDs A, C1, C2, C3, C4, and C5

The hole concentration and radiative recombination rate profiles in the MQW region for LEDs A, C1, C2, C3, C4, and C5 at the current density of 170 A/cm^2^ are presented in Fig. 15b, c, respectively. The data are also collected at the position of 230 μm apart from the left mesa edge. The conclusions here are similar to that for Fig. 6b, Fig. 9b and Fig. 12b, i.e., the adoption of the PNP-AlGaN current spreading layer increases the hole injection, and the hole concentration in the MQW region becomes even more improved once the AlN composition in the n-AlGaN layer increases. We then further calculate and present the EQE and the optical power density in terms of the injection current for the investigated devices in Fig. 16a. Clearly, we can see that the EQE can be improved once the PNP-AlGaN junction is employed. In addition, as the AlN composition in the n-AlGaN layer for the PNP-AlGaN junction increases, the EQE can be further promoted, thanks to the better current spreading, which homogenizes the hole concentration in each quantum well plane as has been shown previously.

Figure 16b investigates the current-voltage characteristics for LEDs A, C1, C2, C3, C4, and C5. The device exhibits a slight increase in the forward operating voltage for LED C1 with the PNP-Al_0.40_Ga_0.60_N junction when compared to the LED A. Meanwhile, the device consumes more forward voltage for LEDs C4 and C5. The observation here is consistent with that in Fig. 7b, Fig. 10b and Fig. 13b, such that the adoption of the PNP-AlGaN junction causes the additional valence band barrier height for holes, which, as a result, increases the forward voltage and even the turn-on voltage (e.g., LEDs C4 and C5). However, it is worth mentioning that the forward operating voltage for LEDs C2 and C3 decreases when compared to LED A. The underlying mechanism is not clear at this moment. However, we tentatively attribute the reduced forward voltage for LEDs C2 and C3 to the hole acceleration effect [35].

**Fig. 17:**
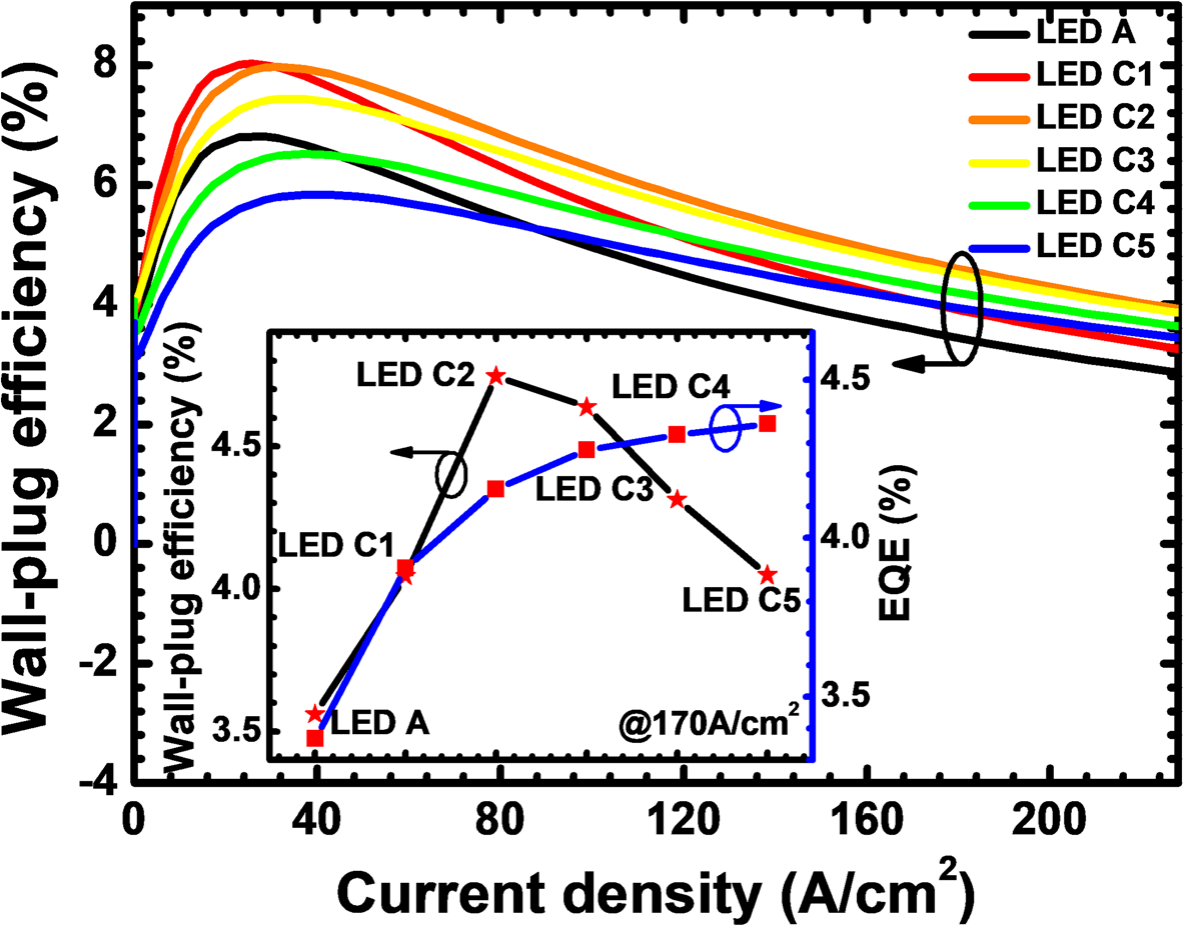
WPE as a function of the injection current for LEDs A, C1, C2, C3, C4, and C5. Inset figure shows the WPE and the EQE for the studied LEDs with various AlN compositions for the n-AlGaN layer at the current density of 170 A/cm^2^

Figure 17 shows the relationship between the WPE and the injection current density for the tested LEDs. We can get that the WPE can be enhanced for all the proposed LEDs especially when the injection current density is beyond 89 A/cm^2^. Insightful study into LED C5 shows that the WPE for LED C5 is lower than that for LED A when the current density is smaller than 89 A/cm^2^. Nevertheless, the WPE for LED C5 overwhelms that for LED A when the injection current density become higher (i.e., > 89 A/cm^2^). As is well known, the current easily gets crowded when the LED device is biased at a high current level. The WPE for LED C5 reflects that the PNP-Al_0.51_Ga_0.49_N junction is indeed effective in improving the current spreading effect. However, considering the additional voltage consumption in the PNP-AlGaN junction, one shall be very careful when setting the AlN composition for the n-AlGaN layer so that the WPE can be maximized according to the inset in Fig. 17.

## Conclusions

To summarize, the PNP-AlGaN junction for DUV LEDs are explored and demonstrated. Assisted by the proposed PNP-AlGaN junctions, the current spreading effect can be improved. The improved current spreading effect is well attributed to increased the vertical resistance and the enhanced horizontal current flow. Moreover, we have also conducted the parametric study to reveal different PNP-junctions on the current spreading effect, the EQE and the WPE. We find that by properly increasing the thickness, the doping concentration, the AlN composition for the n-AlGaN insertion layer, and the number for the PNP-AlGaN junction, the current spreading effect can be improved. On the other hand, we also find that the current spreading effect can indeed enhance the EQE. However, the forward voltage may be increased if the PNP-AlGaN junction is not fully optimized, the cost of which is the reduced WPE. It is also worthy pointing out that the current spreading feature is the cooperative function of the thickness, the doping concentration, the AlN composition for the n-AlGaN insertion layer, and the number for the PNP-AlGaN junction. As a result, there is no unique answer for the best design of the PNP-AlGaN current spreading layer for DUV LEDs. However, we strongly believe that the findings in this work introduce the additional physical understanding to the PNP-AlGaN current spreading layer and the current spreading effect for DUV LEDs. Hence, this work is very useful for the community of optical semiconductor devices.

## Abbreviations

APSYS: Advanced Physical Models of Semiconductor Devices
CBL: Current blocking layer
CL: Current spreading layer
DUV LEDs: Deep ultraviolet light-emitting diodes
EQE: External quantum efficiency
IQE: Internal quantum efficiency
LQW: Last quantum well
MOCVD: Metal-organic chemical vapor deposition
MQWs: Multiple quantum wells
PNP-AlGaN: p-AlGaN/n-AlGaN/p-AlGaN
SRH: Shockley-Read-Hall
WPE: Wall-plug efficiency
